# How do Interpersonal Relationships Relieve Adolescents’ Problematic Mobile Phone Use? The Roles of Loneliness and Motivation to Use Mobile Phones

**DOI:** 10.3390/ijerph16132286

**Published:** 2019-06-28

**Authors:** Rui Zhen, Ru-De Liu, Wei Hong, Xiao Zhou

**Affiliations:** 1 Institute of Psychological Sciences, College of Education, Hangzhou Normal University, No. 2318 Yuhangtang Road, Hangzhou 311121, China; 2 Faculty of Psychology, Beijing Normal University, No. 19 Xinjiekouwai Street, Beijing 100875, China; 3 Department of Psychological and Behavioral Sciences, Zhejiang University, No.148 Tianmushan Road, Hangzhou 310028, China

**Keywords:** problematic mobile phone use, parent–child relationship, teacher–student relationship, loneliness, motivation to use mobile phones

## Abstract

The current study aimed to explore the underlying mechanisms of how interpersonal relationships relieve adolescents’ problematic mobile phone use (PMPU) and to examine the potential mediating roles of loneliness and motivation to use mobile phones. Four thousand five hundred and nine middle school students from four provinces in China were recruited to participate in the investigation. The results showed that the parent–child relationship but not the teacher–student relationship, had a direct and negative effect on PMPU. The parent–child relationship had indirect effects on PMPU through the mediators of loneliness, escape motivation and relationship motivation; the teacher–student relationship had indirect effects on PMPU only through the mediating factors of loneliness and escape motivation. Both parent–child and teacher–student relationships indirectly affected PMPU through a two-step path from loneliness to escape motivation. These findings highlight the more salient role of the parent–child relationship than that of the teacher–student relationship in directly alleviating PMPU and indicate that satisfying interpersonal relationships can buffer adolescents’ PMPU by lowering their loneliness and motivation to use mobile phones.

## 1. Introduction

Mobile phone use has been dramatically increasing in the world in the past decade, as mobile phones facilitate communication without imposing constraints due to physical proximity or spatial immobility [1] and enable users to engage in a wide range of online activities [2]. People have enjoyed the benefits made possible by mobile phones; at the same time, concerns related to mobile phone use have also arisen. One of these problems is problematic mobile phone use (PMPU), which refers to the uncontrolled and excessive use of mobile phones [2]. Studies recently found that PMPU has complex negative effects on individuals’ daily lives and may ultimately lead to depression [3], sleep problems [4] and other negative outcomes [5,6]. Therefore, PMPU has increasingly attracted the attention of researchers and social workers [3,7,8,9] and relevant findings have specifically suggested that adolescents are highly susceptible to PMPU [10] due to their immature cognitive regulation capacity and greater time flexibility [11]. For example, a study reported that the prevalence rate of smartphone addiction in Korean adolescents was 11.4% [12]. Another study on adolescents’ PMPU indicated that 20.5% British adolescents were problematic phone users [13].

Although PMPU prevails among adolescents, not all adolescents report PMPU. Why are some adolescents problematic mobile phone users, whereas others are not? To explain this issue, Billieus’ team proposed the pathway model of PMPU [2,14]. The model suggested three pathways that lead to PMPU, including the need to maintain relationships and obtain reassurance, the desire to communicate with others and establish new relationships and poor control skills [14]. It is obvious that the first two pathways involve interpersonal relationships, which may also exert effects on the third pathway [2]. Thus, interpersonal relationships can be considered a crucial predictor leading to the occurrence of PMPU.

Parents and teachers are the most important adults who provide comfort, guidance and support to adolescents [15,16]. Studies have also indicated that parent–child and teacher–student relationships are two important interpersonal relationships for adolescents and may exert an effect on adolescents’ technology addiction (i.e., problematic internet use) [17]. Social control theory [18] suggests that adherence to conventional institutions, such as those established by parents and teachers, serves as a form of social control. Adolescents under social control may accept supervision and constraints from parents and teachers more readily and the risk of internet addiction would also decrease [17,19]. In addition, a positive relationship with parents and teachers provides adolescents with warmth and security, which may satisfy their offline psychological needs and in turn alleviate their internet addiction [19].

Nevertheless, these studies focused on internet use and few examine the role, as well as the underlying mechanism, of parent–child and teacher–student relationships in PMPU. Furthermore, most studies did not simultaneously examine the effects of parent–child and teacher–student relationships on technology addiction and compare their underlying mechanisms. To advance these issues, the aim of this study was to examine and compare the underlying mechanisms of the effects of parent–child and teacher–student relationships on PMPU.

In fact, parent–child and teacher–student relationships are external environmental factors for adolescents, according to ecological system theory [20]. Importantly, adolescents are not simply passive recipients of environmental factors; they also adapt their behaviors by interacting with the environment [21]. Moreover, environmental factors may exert effects on individual behaviors via individual characteristics, wherein loneliness and motivation to use mobile phones are two important individual characteristics that may predict PMPU [10]. Thus, the current study will examine the mediating roles of loneliness and motivation to use mobile phones in the association between the parent–child/teacher–student relationship and PMPU to elucidate the underlying mechanisms of the two relationships in predicting PMPU.

### 1.1. Potential Mediating Roles of Loneliness and Motivation in Phone Use

Loneliness, the perception of deficiency when one feels that interpersonal relationship networks are smaller or less satisfying than those that are desired [22], is often considered an important risk factor for PMPU [23]. According to Davis’ cognitive-behavioral model of problematic internet use [24], individuals who suffer from loneliness are more likely to have distorted cognitions about the self and the world and develop a strong aversion to and become less satisfied with the real world [25]. Adolescents may resort to using mobile phones to obtain access to the virtual world, which may temporarily relieve them of their loneliness; however, this approach may increase their risk of PMPU [10]. Empirical studies have also found that loneliness can lead to PMPU [23,26,27,28].

Nevertheless, providing positive interpersonal relationships can help to relieve loneliness. For instance, a positive parent–child relationship contributes to the development of interpersonal skills and can satisfy children’s sense of relatedness and social bonding [29] to relieve loneliness [30]. Similarly, positive teacher–student relationships help students feel that they are accepted, which may encourage them to participate positively in group activities [31] and develop a sense of school belonging and general perceived competence [32], thus relieving their feelings of loneliness. Taken together,

**Hypothesis** **1.** *The evidence suggests that loneliness may mediate the role of parent–child and teacher–student relationships in PMPU.*

Another potential mediating factor is motivation to use mobile phones, an internal drive that activates people to intentionally choose mobile phones to gratify psychological needs. Initially, people use phones to build or maintain interpersonal communication. Thus, a common motivation in phone use involves relationship building, which is called relationship motivation [33,34]. With the expansion of the functions of mobile phones, such as its use in playing games and obtaining access to information, the purposes of using mobile phones have also become diverse. Compensatory internet use theory [35] suggests that when suffering from psychological or life problems in the real world, individuals may resort to the internet or to smartphones to escape their suffering. Thus, an important motivation to use mobile phones is escape motivation [36,37]. Studies have indicated that relationship motivation and escape motivation may lead to PMPU [10,34,38]. A potential explanation is that relationship motivation can improve people’s communication with others to satisfy their need for relatedness that cannot be fulfilled in real life, which in turn increases the risk for PMPU. People with escape motivation consider mobile phone use as a dysfunctional coping style when they are confronted with stressful situations in real life, which may also lead to PMPU [10,38].

To alleviate adolescents’ relationship motivation and escape motivation to use mobile phones, the establishment of positive interpersonal relationships in real life and in particular, positive parent–child and teacher–student relationships, should be emphasized. Positive parent–child and teacher–student relationships can not only provide adolescents with warmth and meet their needs for relatedness [29,32,39] but can also improve their social skills [30,31] and thus increase their communication with others in the real world. In such ways, adolescents’ motivation to use mobile phones to engage in virtually mediated communication can be decreased. In addition, positive parent–child and teacher–student relationships can also provide guidance and help to adolescents [40] in handling the problems and difficulties they encounter in the real-world context, which helps to reduce their tendency to escape from these problems and ultimately relieves their escape motivation to use mobile phones. Taken together,

**Hypothesis** **2.** *We propose that parent–child and teacher–student relationships decrease relationship motivation and escape motivation to use mobile phones and in turn relieve PMPU.*

### 1.2. Potential Relation between Loneliness and Motivation in Phone Use

Although both loneliness and motivation to use mobile phones may uniquely mediate the buffering role of parent–child and teacher–student relationships in PMPU, it is still unclear whether loneliness and motivation to use mobile phones play a combined mediating role. In fact, loneliness can lead to adolescents’ deficits in social interaction [10], decrease their competency in interactions and thus result in their avoidance of social interaction [41]. In addition, as social interaction in the real world is usually synchronous, anxiety, especially for those who feel less confident in their social skills, will be induced [42]. In addition, deficits in real-world social interaction make it difficult for lonely people to build positive interpersonal relationships. To meet the need for relatedness and to avoid the distress brought by real-world interaction, lonely people prefer mediated interaction to real-world interaction because the former is characterized by anonymity and asynchronous features [43]. Mobile phone use is one approach to mediated interaction. Thus, loneliness may activate relationship motivation and escape motivation to use mobile phones [10]. Regarding the potential mediating roles of loneliness and motivation to use mobile phones in the association between parent–child/teacher–student relationship and PMPU,

**Hypothesis** **3.** *It is likely that the parent–child/teacher–student relationship will predict PMPU by the multiple indirect path from loneliness to motivation to use mobile phones.*

Although several potential predictions have been advanced to describe the relationships among the parent–child/teacher–student relationship, loneliness, motivation to use mobile phones and PMPU, the predictive power of these predictions has not been simultaneously evaluated. Moreover, previous studies have mainly focused on the unique roles of the parent–child/teacher–student relationship, loneliness and motivation to use mobile phones in PMPU but their combined role remains unclear. Furthermore, it is unknown whether the underlying mechanisms of the parent–child/teacher–student relationship on PMPU through relationship and escape motivation are the same. To fill these gaps, this study will examine and compare the underlying mechanisms of the parent–child and teacher–student relationships on PMPU via loneliness and relationship/escape motivation.

## 2. Method

### 2.1. Participants and Procedures

In November 2017, we recruited 4509 middle school students to participate in our investigation and the overall response rate was 89.83%. These students were in grades 7, 8, 10 and 11 and they came from two schools in Beijing (*N* = 1927), one in Anhui Province (*N* = 1378), one in Fujian Province (*N* = 287) and one in Hunan Province (*N* = 917), respectively. Province participation ranged from Fujian Province 6.37% to Anhui Province 42.74%. We did not assess middle school students in grades 9 or 12 because they were occupied by preparations for the senior middle school or college entrance examination. Of these participants, there were 2265 (50.23%) boys and 2043 (45.31%) girls; 201 (4.46%) students did not report sex. On average, the participants were 14.05 years old (SD = 1.82), ranging from 10 to 19 years old.

This study was approved by the principals of the participating schools. There were no exclusion criteria and everyone in grades 7, 8, 10 and 11 who attended school on the day of the investigation was eligible to participate. Before the investigation, researchers informed them of the research purpose and the voluntary nature of participation and participants were free to withdraw from the survey at any time. Written consent forms were provided to both the participants and their teachers. In a classroom setting, participants were asked to provide demographic information and to use scales to rate their relationships with parents and teachers, their motivation for using mobile phones, their loneliness and their PMPU. After the students completed the survey, researchers told them that school psychologists or teachers were available to provide any psychological/counseling services if needed.

### 2.2. Measures

#### 2.2.1. Parent–Child Relationship

Adolescents assessed their perceived relationship with their parents by using 10 items from the Family Adaptability and Cohesion Evaluation Scales [44,45]. The scale had two subscales involving father-child and mother-child relationships and each item was rated on a 5-point Likert scale (1 = not true at all, 5 = extremely true). Sample items included “When in trouble, my father/mother and I will support each other,” “I have common hobbies and interests with my father/mother.” The scale exhibited acceptable reliability (*α_father-child_* = 0.74, *α_mother-child_* = 0.71) in the current research. In this study, we used the mean score of adolescents’ perceived relationships with their fathers and mothers to indicate their overall relationship with parents.

#### 2.2.2. Teacher–Student Relationship

A teacher–student relationship scale was adopted to measure how adolescents perceived their relationships with teachers. Based on the original student–teacher relationship scale [46] for teachers’ perceptions of their relationship with children from kindergarten to grade 3, an inventory for teachers’ perceptions of their relationships with students from grade 4 through junior high school [47] and the applicability of a Chinese scale for students’ perceptions of their relationships with teachers [48], we revised and developed a teacher–student relationship scale that included subscales pertaining to closeness (5 items), instrumental help (4 items), satisfaction (4 items) and conflict (5 items). Adolescents responded to each item using a 5-Likert scale (1 = not true at all, 5 = extremely true) and sample items included “I will ask for my teachers’ help when I have problems” and “My teachers often criticize or punish me.” The revised scale demonstrated adequate reliability (0.73 < *αsubscales* < 0.82) and acceptable construct validity [χ^2^/df = 2207.72 (127), comparative fit index (CFI) = 0.93, Tucker-Lewis index (TLI) = 0.91, root mean square error of approximation (RMSEA) (90% CI) = 0.068 (0.066–0.070)] in the current study.

#### 2.2.3. Loneliness

We used a loneliness scale by Asher, Hymel and Renshaw [49] to assess adolescents’ feelings of loneliness or perceived peer status. The scale consisted of 16 items and each of them was rated on a 5-point Likert scale (1 = not true at all, 5 = always true). Sample items included “I don’t have anyone to play with” and “I am lonely.” The scale exhibited good reliability in previous research [49] as well as in the current research (*α* = 0.90).

#### 2.2.4. Motivation to Use Mobile Phones

We used a scale by Kim [10] to assess adolescents’ motivation to use mobile phones. The scale consisted of two subscales: escape motivation (6 items) and relationship motivation (3 items). Sample items included “I use mobile phones to forget about school work” and “I use mobile phones to feel closer to family and friends.” Adolescents responded to all items on a 5-point Likert scale (1 =not true at all, 5 =extremely true). The scale had good reliability (*α_escape_* = 0.87, *α_relationship_* = 0.86) in the current research.

#### 2.2.5. Problematic Mobile Phone Use

A short 10-item scale by Foerster, Roser, Schoeni and Röösli [50] was used to measure adolescents’ problematic mobile phone use. The shortened scale involves five aspects related to addiction symptoms, including craving, negative life consequences, withdrawal, loss of control and peer dependence. Sample items included “I feel anxious if I have not checked for messages or switched on my mobile phone for some time.” Each item was rated on a 5-point Likert scale (1 = not true at all, 5 = extremely true) and a higher aggregated score indicates a higher degree of mobile phone dependence. The scale was suggested as a suitable instrument for adolescents and had good reliability both in previous research [50] and in the current research (*α* = 0.83).

### 2.3. Data Analytical Strategies

Descriptive analyses and Pearson correlations were calculated for the main measures. Statistical analyses were conducted using Mplus 7.0 software (Muthén & Muthén, Los Angeles, CA. US) [51]. Little’s “Missing Completely at Random” (MCAR) test was conducted to examine the pattern of missing data. The results [χ^2^(120) = 162.814, *p* < 0.05] revealed that data were not missing completely at random, thus we used robust maximum likelihood (MLR) estimations for missing data when running models [52].

To evaluate model fit, chi-square values, the comparative fit index (CFI), the Tucker-Lewis index (TLI), the root mean square error of approximation (RMSEA) and the standardized root mean residual (SRMR) were used. The cutoffs for model acceptability were a CFI and TLI greater than or equal to 0.90 and an RMSEA and SRMR less than or equal to 0.08.

We followed a two-step procedure to examine the multiple mediating roles of loneliness and relationship/escape motivation in the association between parent-/teacher–student relationship and PMPU. First, a direct effect model was built to assess the direct effect of the parent-/teacher–student relationship on PMPU. Second, we put loneliness and relationship/escape motivation as mediators of the direct path and added predictive paths between these mediators. Furthermore, to test the significance of the indirect effects, we conducted bias-corrected bootstrap tests with a 95% confidence interval.

## 3. Results

### 3.1. Descriptive Statistics and Correlations between Main Measures

Table 1 reports the results of descriptive statistics and correlations between the main measures. The parent–child relationship was significantly related to loneliness, escape motivation, relationship motivation and PMPU. The teacher–student relationship was also significantly associated with the other measures except for relationship motivation. Loneliness had a significant relationship with the other measures except for relationship motivation. Both escape and relationship motivation had significant and positive relationships with PMPU.

### 3.2. Mediating Roles of Loneliness and Motivation to Use Mobile Phones

We firstly built a model to examine the direct predictions of parent–child and teacher–student relationships on PMPU. The results found that the model fit the data completely [χ^2^(0) = 0, CFI = 1.00, TLI = 1.00, RMSEA (90% CI) = 0.00, SRMR = 0.00] and path analysis showed that parent–child and teacher–student relationships negatively predicted PMPU (*β* = −0.17, *p* < 0.001; *β* = −0.10, *p* < 0.001), respectively.

**Figure 1:**
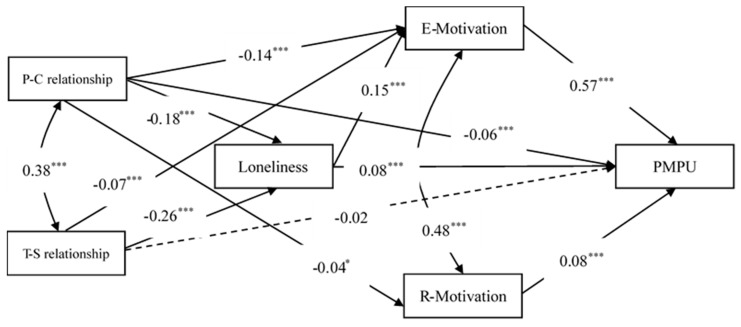
The multiple indirect effects model of parent–child/teacher–student relationship on problematic mobile phone use. P–C relationship = Parent–child relationship, T–S relationship = Teacher–student relationship, E-Motivation = Escape motivation, R-Motivation = Relationship motivation, PMPU = Problematic mobile phone use. Dashed line represents a non-significant coefficient, single arrows represent predicting paths from one variable to another and bi-arrows represent correlations between dimensions of one variable. Predicting paths without a mediator indicate the direct effect(s), whereas with a mediator(s) the indirect effect(s). * *p* < 0.05, *** *p* < 0.001.

Next, we placed loneliness and motivation to use mobile phones (e.g., escape and relationship motivation) between the parent–child/teacher–student relationship and PMPU and built a multiple indirect effects model (see Figure 1). Given the non-significant associations between the teacher–student relationship/loneliness and relationship motivation, the paths from the teacher–student relationship/loneliness to relationship motivation were not added in this model. This model showed good fit indices [χ^2^(2) = 1.60, CFI = 1.00, TLI = 1.00, RMSEA (90% CI) = 0.00 (0.00–0.028), SRMR = 0.005]. The model results found that the parent–child relationship but not the teacher–student relationship, had a direct and negative association with PMPU. The parent–child relationship had indirect prediction on PMPU via three mediators: loneliness, escape motivation and relationship motivation. The teacher–student relationship had indirect prediction on PMPU via two mediators: loneliness and escape motivation. The parent–child/teacher–student relationship had indirect prediction on PMPU through a two-step path from loneliness to escape motivation.

Furthermore, we examined the significance of the mediating effects above by using a bias-corrected bootstrap test. As shown in Table 2, the mediating effects were all significant, indicating that the parent–child relationship directly predicted PMPU and could also predict PMPU via loneliness, escape motivation and relationship motivation. In contrast, the teacher–student relationship only indirectly predicted PMPU via loneliness and escape motivation. Both the parent–child and the teacher–student relationships predicted PMPU by loneliness via escape motivation.

## 4. Discussion

To our knowledge, this study is among the first to simultaneously examine and compare the underlying mechanism of the roles of parent–child and teacher–student relationships in predicting PMPU. The findings indicated that the parent–child relationship could predict PMPU via loneliness and relationship/escape motivation to use mobile phones but the teacher–student relationship could only predicted in PMPU via loneliness and escape motivation rather than via relationship motivation. The findings provide evidence that the underlying mechanisms how the parent–child and teacher–student relationships link to PMPU show similarities and differences.

Specifically, the parent–child relationship but not the teacher–student relationship, directly and negatively predicted PMPU. Although studies have indicated that both parents and teachers supervise adolescents’ technological addiction [17,19], these studies focused on adolescents’ internet addiction. Compared to the internet, mobile phones are becoming necessary devices for communication between parents and adolescents. A good parent–child relationship not only provides adolescents with a warm environment and positive communication to satisfy their need for relatedness [53] but also helps in the supervision of adolescents’ daily behaviors. These factors all serve to protect adolescents from PMPU. In contrast, although some studies have indicated that a good teacher–student relationship provided better supervision and guidance with respect to students’ phone use behaviors [17,19], most middle school students in China are currently forbidden to take mobile phones to schools but a good teacher–student relationship cannot exert a direct influence on students’ PMPU outside of school.

Nevertheless, both positive parent–child and teacher–student relationships negatively predicted PMPU by adolescents’ escape motivation. In fact, parents and teachers are the most important and authoritative adults in the lives of adolescents [15,16]. Hence, a warm relationship with them provides a safe environment in which adolescents can make brave excursions into the world (to play, work, learn, discover, create, etc.), since they know that they can return to these adults for comfort, reassurance and assistance when they encounter difficulties along the way [54]. Adolescents are encouraged to explore the world and to address problems positively, rather than to escape from reality. The decline in escape motivation can further encourage adolescents to positively engage in real-world social activities rather than in online or virtual activities, which in turn lowers their potential PMPU.

In addition, both positive parent–child and teacher–student relationships negatively predicted PMPU by adolescents’ sense of loneliness or through a multiple mediating path from loneliness to escape motivation. Positive social relationships can facilitate individuals’ interpersonal interactions and help them obtain more social support [55] and individuals are more likely to experience feelings of intimacy and being loved, which can relieve their perceived loneliness [56,57]. Loneliness drives adolescents to resort to browsing the internet or playing games on their mobile phones to ease their negative emotions, leaving them vulnerable to developing PMPU. In contrast, adolescents’ PMPU will be relieved if they feel less lonely and have positive social relationships. In addition, previous studies have indicated that relieving loneliness can increase individuals’ willingness to interact with others in the real or offline world and lower their evasive tendencies [10,43], thus reducing the frequency of mobile phone use and resulting in less PMPU.

One interesting finding was that the parent–child relationship, rather than the teacher–student relationship, had an indirect negative association with PMPU via decreasing adolescents’ relationship motivation. Although both parents and teachers are important figures, parents are the primary sources of relatedness and attachment for adolescents in their explorations of the world [29] and compared with teachers, they play a more durable and important role in adolescent development. In addition, parents are considered to be the first guide to children in establishing relationships [58]. Specifically, they can provide beneficial advice and guidance to help adolescents build better interpersonal relationships [59], thereby contributing to their relationship satisfaction in the real world. This helps to decrease their motivation to use mobile phones to fulfill their need for interpersonal relationships and thus reduces their probability of engaging in PMPU.

Furthermore, this study found that neither parent–child nor teacher–student relationships had significant prediction on PMPU by loneliness via relationship motivation. This finding may be due to the non-significant relationship between loneliness and relationship motivation, which is inconsistent with previous findings [10] and our assumption. A potential explanation is that lonely individuals tend to show social withdrawal [60,61], thus enabling them to avoid feeling shy or nervous when interacting with others [62]. This approach may prevent lonely individuals from engaging in any interpersonal communication, whether online or offline. Therefore, in this study, loneliness showed a non-significant association with relationship motivation to use mobile phones.

Some limitations should be noted. First, some social-demographic variables such as grade, gender, school type and family social economic status and so forth, are potential factors for PMPU but we did not take them into consideration. Researchers can include them and examine their unique roles. Second, although parents and teachers are the most important adults in adolescents’ lives [15,16], peer relationships may also play a crucial role in adolescents’ addictive behaviors [63] and future studies can simultaneously examine and compare the unique roles of peers, parents and teachers in PMPU. Third, the cross-sectional design of this study indicates that causal descriptions among main variables are only based on theoretical assumptions. Fourth, our participants focused on adolescents in middle schools, so caution should be exercised in the generalization of these findings to other samples.

## 5. Conclusions

This study first examined and compared the underlying mechanisms of the association between parent–child and teacher–student relationships and PMPU in an adolescent sample and found that the two influencing mechanisms had both similarities and differences. To be specific, the parent–child relationship but not the teacher–student relationship, had a direct and negative effect on PMPU. The parent–child relationship had indirect effects on PMPU through the mediators of loneliness, escape motivation and relationship motivation; the teacher–student relationship had indirect effects on PMPU only through the mediating factors of loneliness and escape motivation. Both parent–child and teacher–student relationships indirectly affected PMPU through a two-step path from loneliness to escape motivation. These findings indicated that parents played a more important role than teachers in relieving adolescents’ PMPU. From a theoretical perspective, the findings contribute to the extension of the PMPU theory from the standpoint of interpersonal relationships. From an intervention perspective, our findings suggest that parents and teachers need to help adolescents develop positive communication habits and establish positive parent–child and teacher–student relationships to relieve adolescents’ perceptions of loneliness or escape/relationship motivation, thus further reducing their risk of PMPU.

## Figures and Tables

**Table 1:** Means, standard deviations and correlations among main variables.

Main Variables	M (SD)	1	2	3	4	5
1. Parent–child relationship	3.43 (1.13)	1.00				
2. Teacher–student relationship	3.55 (0.63)	0.38 ***	1.00			
3. Loneliness	1.92 (0.69)	−0.27 ***	−0.32 ***	1.00		
4. Escape motivation	2.70 (1.06)	−0.21 ***	−0.16 ***	0.20 ***	1.00	
5. Relationship motivation	2.49 (1.17)	−0.04 *	0.003	−0.008	0.47 ***	1.00
6. PMPU	2.36 (0.79)	−0.21 ***	−0.16 ***	0.22 ***	0.64 ***	0.35 ***

Note: * *p* < 0.05, *** *p* < 0.001.

**Table 2:** Bias-corrected bootstrap test on mediating effects.

PMPU	Parent–Child Relationship		Teacher–Student Relationship	
	95% CI	*β*	95% CI	*β*
*One-step mediation*				
Indirect via loneliness	[−0.020 ~ −0.009]	−0.015 ***	[−0.029 ~ −0.013]	−0.021 ***
Indirect via escape motivation	[−0.103 ~ −0.058]	−0.081 ***	[−0.059 ~ −0.019]	−0.039 ***
Indirect via relationship motivation	[−0.006 ~ <0.000]	−0.003 *		
*Two-step mediation*				
Indirect via loneliness and escape motivation	[−0.020 ~ −0.011]	−0.015 ***	[−0.027 ~ −0.016]	−0.022 ***

Note: * *p* < 0.05, *** *p* < 0.001.
